# Acute and subacute toxicity studies of a poly herbal formulation used for diabetes

**DOI:** 10.12669/pjms.38.6.5928

**Published:** 2022

**Authors:** Aymen Owais Ghauri, Ejaz Mohiuddin, Tayyeba Rehman, Hk. Sheraz Muhammad Siddiqui

**Affiliations:** 1 Aymen Owais Ghauri, PhD Eastern Medicine (fellow) Department of Eastern Medicine, Jinnah University for Women, Karachi, 74600, Pakistan. Faculty of Eastern Medicine, Hamdard University, Karachi, Pakistan; 2 Dr. Ejaz Mohiuddin, MBBS PhD Eastern Medicine Faculty of Eastern Medicine, Hamdard University, Karachi, Pakistan; 3 Tayyeba Rehman, M.Phil. Phytomedicine University College of Conventional Medicine, Faculty of Medicine and Allied Health Sciences, The Islamia University of Bahawalpur, Bahawalpur (63100), Pakistan; 4 Dr. Hk. Sheraz Muhammad Siddiqui, PhD Eastern Medicine Faculty of Eastern Medicine, Hamdard University, Karachi, Pakistan

**Keywords:** PHF-dia, Poly herbal formulation, Diabetes, Toxicity

## Abstract

**Objectives:**

PHF-dia (Poly Herbal Formulation Diabetes) is a polyhedral formulation possessing antihyperglycemic and antihyperlipdimic effects. This study aims to assess acute and sub-acute toxicity of PHF-dia in rats.

**Methods:**

This is an experimental study conducted in two different phases. Acute toxicity was conducted for 14 days and sub-acute toxicity was conducted for 28 days. Both studies were conducted in animal house of Jinnah University for Women, Karachi, Pakistan. Acute toxicity was evaluated in vivo with single time oral administration of 400 mg/kg and 2000 mg/kg doses for two weeks. Sub-acute toxicity was investigated with the application of repeated doses of 150 mg/kg/day, 250 mg/kg/day and 500 mg/kg/day for 28 days.

**Results:**

Acute toxicity study results showed no toxic symptoms, behavioral changes or death in rats up to 2000 mg/kg. Therefore, LD_50_ of oral toxic dose must be more than 2000 mg/ml. Similarly, sub-acute toxicity studies confirmed the safety of PHF-dia and showed no clinical symptoms nor biochemical or histological variation in rats treated with 150 mg/kg, 250 mg/kg and 500 mg/kg compared to the control group (p <0.05).

**Conclusion:**

This indicates safe nature of PHF-dia for the further clinical trials. However, mechanism of action of PHF-dia is not fully understood.

**Abbreviations:**
ANOVA: Analysis of variance
ALT: Alanine transaminase
AST: Aspartate transaminase
EDTA: Ethylene diamine tetra-acetic acid
H & E: Haematoxylin and Eosin
HDL: High density lipoprotein
LD_50_: Median lethal dose
LDL: Low density lipoprotein
PLT: Platelet count
RBC: Red blood cell
SPSS: Statistical package for social science
VLDL: Very low-density lipoprotein
WBC: White blood cell
PHF-dia: (Poly Herbal Formulation Diabetes)

## INTRODUCTION

Diabetes Mellitus (DM) is a metabolic disease increasing immensely all over the world. Carbohydrates, proteins and lipids metabolism disturbs that causes many complications. DM is being presumed as the largest worldwide non-contagious disease by year 2025. It is also hypothesized that developing world will be more prone to DM.^1^,^2^ There is lack of systematized health care system in developing countries.^3^ Only a tiny population has access to the modern medicine while other mostly relay on native herbs having hypoglycemic potential.^4^

A poly herbal formulation (PHF-dia) consisted of *Syzigium cumini* (seeds), *Holarrhena antidysenterica* (seeds), *Citrullus collocynthis* (fruit) having reported antioxidant, hypoglycemic and hypolipidemic effects in streptozotocin induced diabetic rats.^4^ All the plants included in PHF-dia have traditional evidence based ant diabetic effects.^5^,^6^ A common belief prevailed is about the harmlessness of natural herbal medicines due to which safety profile and toxicity studies of traditional herbal medicines are scarce. Therefore, we planned this study to investigate acute and sub-acute toxicity of PHF-dia. Ex-vivo, in vitro and animal models can be used as test systems. However, for toxicity evaluation laboratory animals are more suitable than other studies.^7^ Male rats were used because they are more sensitive to assess acute and sub-acute toxicity.^8^ The primary objective of this study was to access acute and sub-acute toxicity for safe application of PHF in future clinical trials.

## METHODS

This is an experimental study conducted in two different phases. Acute toxicity was conducted for 14 days and sub-acute toxicity was conducted for 28 days. Both studies were conducted in animal house of Jinnah University for Women, Karachi, Pakistan.

Plant parts namely *Syzigium cumini* (seeds), *Holarrhena antidysenterica* (seeds), *Citrullus collocynthis* (fruit) were purchased as a dried herb from local market of Bahawalpur, Pakistan and were authenticated in the Department of Botany, Islamia University of Bahawalpur. The voucher specimens were placed at the herbarium of IUB (Voucher no 2201/L.S).Extracts of selected plants were made according to methodology of Abbasi et al 2018.^9^ The poly herbal formulation was prepared by taking specific ratios of extracts of selected plant parts based on method of Pari L & Saravanan R et al.^10^

This study was carried out on Wistar male rats. Average weight of Wistar rats was 145-250g. They were reserved in normal atmosphere (25°C, 12h/12h light/dark cycle) with standard diet and water *ad libitum*. Acclimatization was done for one week prior to study. Study design and procedures were performed according to National Toxicology Program 2016. Prior ethical permission was obtained from the animal ethical committee of Jinnah Women University of Karachi (Ethical approval number: EC/EMS/0252020). Animals were grouped in to three and four groups for acute and sub-acute toxicity studies respectively. For acute toxicity studies, two dosages were tested i.e., 400mg/kg and 2000mg/kg while for sub-acute toxicity studies, 150mg/kg, 250mg/kg and 500mg/kg dosages were tested. General toxicity guidelines for natural traditional medicines are followed.^7^

### Acute toxicity study test

For this study male Wistar rats were used and were kept fasted for 24 hours. A single dose of PHF 2000 mg/kg was administered to each rat. They were kept under observation for first 30 minutes following dose. They were then observed during first 24 hour and for 72 h respectively. The parameters studied were body weight, drowsiness, lacrimation, nasal bleeding, paralysis, piloerection, salivation, skin, utilization of food, water and death.^11^

### Sub-acute toxicity study

The animals were separated in four groups, each having six animals. One group was set as control. In control group normal food and water was given. Other three groups received the doses of 150mg/kg b.w/day, 250mg/kg b.w/day and 500mg/kg b.w/day respectively. The weight of animal was measured weekly and their behavioral changes, morphological changes were also observed. The animals were anaesthetized on 28^th^ day of treatment by introducing 5ml/kg of 1% solution of chloralose in 25% urethane (w/v) intra peritoneal. For the collection of blood sample cardiac puncture was performed. Blood sample was collected in EDTA tubes and heparinized tubes it was then sent for hematological and biochemical analysis.^12^,^13^

### Biochemical Analysis

Samples of blood were analyzed by CBC machine. The contents include; RBCs count, PLT count, WBCs count, Hb, monocytes, lymphocytes, basophils, neutrophils and eosinophil. Serum urea, uric acid, creatinine, cholesterol, triglycerides, HDL, VLDL, ALT, AST, acid phosphatase, and alkaline phosphatase were calculated.

### Histopathological Analysis

The heart, liver, adipose tissues and kidney sections were fixed in 10% neutral buffered formalin overnight at room temperature. Histopathology was performed by the method of Loha et al 2019.^14^

### Statistical Analysis

Data were presented as mean ± SEM. Statistical analysis was performed by using SPSS version 20. One way ANOVA followed by LSD *post hoc* test. The P value of less than 0.05 was considered significant.

## RESULTS

Active phytoconstituents might be responsible for hypoglycemic and hypolipidemic activity in PHF-dia constitutes of Jambosine, gallicacid, ellagicacid, corilagin, 3,6 hexahydroxydiphenoyl-glucose, 4,6-hexahydroxydiphenoylglucose, 1-galloylglucose, 3-galloylglucose, quercetin, β-sitoterol from Syzigium cumini. Cucurbitacin, flavonoids, alkaloids, saponins and phenolic acids, from *C.colocynthis* which have proven anti-diabetic, antioxidant, and insulinotropic effects. Steroidal alkaloids, flavonoids, triterpinoids phenolic acids, tanins resin, coumarins, ergostenol Conimine (C_22_H_36_N_2_) Antidysentericine (C_23_H_36_N_2_O) by regulating alpha glucosidase enzyme thus maintaining post prandial glucose surge.

**Fig.1:**
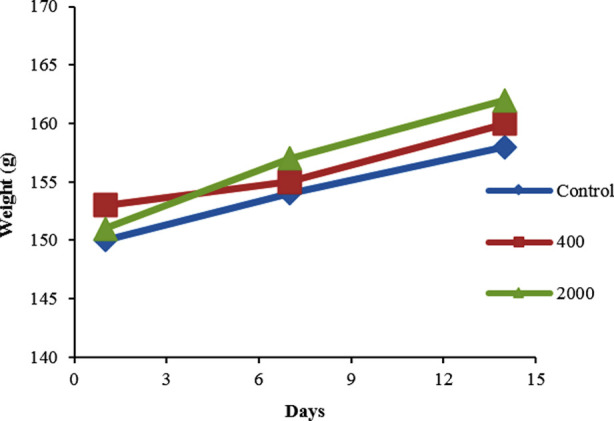
Effect of treatment of PHF on body weight evolution of rats. There was no statistical difference between control group and PHF different dosages p≤0.05.

Results of acute toxicity study indicated safe nature of PHF-dia at doses of400 mg/kg and 2000mg/kg. No death or behavioral change was observed in the treated groups at tested doses. So, the LD_50_ of the PHF could be greater than 2000mg/kg body weight.Fig.1 describes weight evolution of rats during acute toxicity studies of PHF. There was no statistical difference of body weights of control and tested groups (400 mg/kg and 2000 mg/kg).

**Fig.2:**
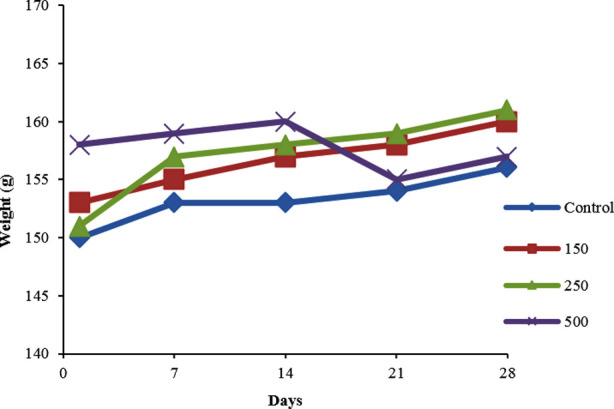
Effect of treatment of PHF on body weight evolution of rats. There was no statistical difference between control group and PHF different dosages p≤0.05.

During sub-acute toxicity study, there were no gross pathological changes in the liver, heart, adipose tissues, pancreas and kidneys of the control and experimental groups. Throughout the sub-acute study, no death or toxic effects were detected on experimental rats (125 mg/kg, 250 mg/kg and 500 mg/kg). There was no significant difference (P > 0.05) observed in the mean rat body weights between control and treated groups (Fig.2).

In the sub-acute toxicity study RBC, WBC, PLT, HB, neutrophils, lymphocytes, eosinophils, monocytes, basophils of the experimental groups (125 mg/kg, 250mg/kg and 500 mg/kg) were within the reference range for rats.^15^ The hematological values in the control group and PHFgroups were not significantly different (Table-I).

**Table-I:** Hematological parameters of rats during sub-acute toxicity studies.

Parameters	Control	125mg/kg	250mg/kg	500mg/kg
WBCs (10*3/ul)	6.9 ± 0.071	6.5 ± 0.067	6.5 ± 0.45	6.4 ± 0.06
RBCs (10^6^/ul)	6.3 ± 0.21	6.8 ± 0.21	6.5 ± 0.05	6.4 ± 0.08
PLT (10*3/ul)	652 ± 0.30	648± 0.34	655.7 ± 0.10	648.5± 0.16
Hb (g/dl)	12.7 ± 0.01	13.4 ± 0.05	12.7 ± 0.15	12.9 ± 0.05
Neutrophils	22.8± 0.01	22.7 ± 0.04	22.7 ± 0.01	23 ± 0.04
Basophils	0.125 ± 0.01	0.15 ± 0.03	0.175 ± 0.01	0.162 ± 0.01
Lymphocytes	73.2 ± 0.12	74.5 ± 0.19	73.5 ± 0.04	75 ± 0.05
Monocytes	0.82 ± 0.03	0.75 ± 0.01	0.78 ± 0.01	0.83 ± 0.02
Eiosinophils	2.2 ±0.001	2.1±0.04	2.1±0.05	2.1±0.02

There was no statistical difference between control group and PHF different dosages p≤0.05.

In the sub-acute toxicity study, the biochemical parameters, such as urea, creatinin, uric acid, ALT, AST, acid phosphatase, alkaline phosphatase, triglycerides, HDL, LDL of experimental groups were within the reference range for rats.^15^ The biochemical values in the control group and PHF groups were not significantly different (Table-II).

**Table-II:** Biochemical parameters of rats during sub-acute toxicity studies.

Parameters	Control	150mg/kg	250mg/kg	500mg/kg
Blood Urea (mg/dl)	44.8± 0.189	44.3 ± 0.78	44.3 ± 0.45	44 ± 0.12
Creatinine (mg/dl)	0.467 ± 0.007	0.49± 0.007	0.495 ± 0.007	0.49 ± 0.17
Uric acid (mg/dl)	3.34 ± 0.89	3.34 ± 0.160	3.35 ± 0.19	3.34 ±0.017
Acid phosphatase (U/L)	27.4 ± 0.071	29.3 ± 0.067	29.5 ± 0.45	29.7 ± 0.06
Alkaline phaosphatase(U/L)	63.1 ± 0.160	64.3 ± 0.067	64.2 ± 0.45	64 ± 0.189
ALT (µl/ml)	36.21 ± 0.21	35.23 ± 0.21	34.21± 0.05	35.21 ± 0.08
AST (µl/ml)	64.4 ± 0.30	36.17± 0.34	40.15 ± 0.10	39.34 ± 0.16
Triglycerides (mg/dl)	26.6 ± 0.30	26.3 ± 0.01	24.9 ± 0.10	27.8 ± 0.30
HDL (mg/dl)	25.5 ± 0.189	24.7± 0.05	24.3 ± 0.5	24.1 ± 0.30
LDL (mg/dl)	10.3 ± 0.05	10.3 ± 0.4	10.6 ± 0.30	10.2 ± 0.189

There was no statistical difference between control group and PHF different dosages p≤0.05.

**Fig.3:**
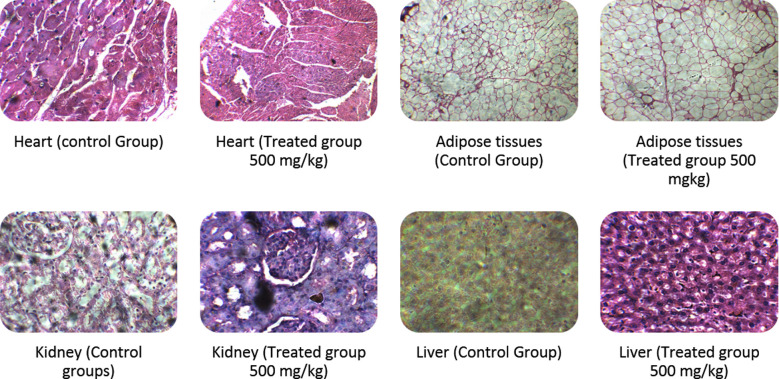
Histopathology of rats during sub-acute toxicity studies.

In the histopathological studies hematoxylin and eosin stain technique was used, the sections of the kidneys, liver, adipose tissues, and heart of treated rats showed normal general structure with no significant difference as compared to control. The lense used to capture these pictures from histopathological slides was of 10 magnifications hence is of 100 scale bars (Fig.3).

## DISCUSSION

This study evaluates the toxicity profile of PHF-dia in rats in two phases. First phase determines acute toxicity while 2^nd^ phase assesses sub-acute toxicity. Changes in the body weight is used as an index of toxicity evaluation.^16^ PHF-dia might be considered relatively safe on acute exposure as it showed no toxic effect or body changes in rats. Sub-acute toxicity study analyses toxicity caused by repeated oral dosing of a substance for 28 days in rodents. This study gives evidence of any change in organ morphology, hematological changes or biochemical parameters in the organism. This study could be used as the basis for the determination of the toxicity effects.^14^

Hematological parameters examination can be used to identify any harmful effect of foreign compounds including in poly herbal formulation on blood.^17^ Biochemical parameters such as urea, creatinine, uric acid, ALT, AST, acid phosphatase, alkaline phosphatase, triglycerides, HDL and LDL etc. have substantial roles as a marker. Liver and kidney function assessment is of main significance to assess the toxic properties of poly herbal formulations and drugs.^18^ Urea measurement is an indicator of kidney function. It could be raised in several acute and chronic renal disorders.^19^ Normal range of serum urea in rats is 15-45mg/dl.^2^ Creatinine is used as a marker of glomerular filtration rate.^20^ Normal value of creatinine in rats is 0.2-0.8 mg/dl.^21^ ALT is a sensitive marker for checking any liver cell damage^22^ while AST is present in RBCs, heart, kidney and skeletal muscles.^23^ PHF-dia showed no toxic effects on hematological and biochemical parameters. The study indicates the safe nature of PHF-dia as it is confirmed by former studies that 10 ± 0.04 mg/mL is confirmed dose exhibiting α-glucosidase potential in jamun seed extract,0.1mg/ml of the profuse extracts of *citrus colocynthis* produced remarkable hypoglycemic activity by sensitizing insulin production from pancreas whereas,300 mg/week is the safe dose studied in literature for ant diabetic purpose. So, it can be used safely for treatment of diabetes and might be proved as an effective alternative option for control of hyperglycemia and hyperlipidemia.

### Limitation of the study

Study indicates a safe alternative choice of treatment for Diabetes. However, mechanism of action of PHF-dia is not fully understood.

## CONCLUSION

The acute toxicity study of PHF-dia showed safe nature of PHF-dia up to 2000 mg/kg. No morbidity and mortality were observed at tested doses. So, oral LD_50_ of PHD-dia will be more than 2000mg/kg. Meanwhile, sub-acute toxicity studies of PHF-dia did not produce adverse effects on body weight of rodents, organ weights & morphology, hematological and biochemical parameters. Moreover, histopathological studies indicate no significant difference between control and treated animal heart, liver, adipose tissues and kidney sections. These studies indicate the safe nature of PHF-dia for the further testing of herbal formulation in human clinical trials.

### Authors Contribution:

**AOG** conceived, data collection designed, manuscript writing and is responsible for integrity of the study.

**EM** did review and final approval of manuscript.

**TR** did statistical analysis & critical review of manuscript.

**DHSMS** facilitated research word and did editing of manuscript.
